# Effects of parenting practices on sexual risk-taking among young people in Cameroon

**DOI:** 10.1186/1471-2458-13-616

**Published:** 2013-06-28

**Authors:** Estelle M Sidze, Barthelemy Kuate Defo

**Affiliations:** 1 African Population and Health Research Center (APHRC), P.O. Box 10787–00100, Nairobi, Kenya; 2 Institut de Recherche en Santé Publique & Centre de Recherche du Centre Hospitalier Universitaire, Université de Montreal, C.P. 6128, Succursale Centre-ville, Montréal, H3C 3J7, Canada

**Keywords:** Parenting practices, Sexual risk-taking, Life course, Africa, Cameroon

## Abstract

**Background:**

There is scanty evidence regarding the impact of parenting practices on young people’s sexual risk-taking in sub-Saharan Africa. Moreover, the extent to which such practices have enduring consequences on adolescents and young adults is little documented. This study uses repeated measures of parent–child relationships, parental monitoring, and parent–child communication about sexual matters to shed some light in these two areas.

**Methods:**

The analysis is based on time-dependent retrospective data on parenting practices which were retrieved from the Cameroon Family and Health Survey (CFHS). The study sample includes 447 sexually active and unmarried individuals aged 15–24 years old. Correlation analysis and multivariate logistic regressions are used.

**Results:**

Young males and females reported high levels of parental monitoring, moderate quality of parent–child relationships and low levels of parent–child communication on sexual matters. This study substantiates that the higher the quality of parent–child relationships, the lower the odds of young males having multiple sexual partners (0.63, *p* < 0.05), and the lower the odds of young females being sexually active (0.52, *p* < 0.10) or of having multiple sexual partners (0.64, p < 0.10) or of having occasional sexual partners (0.51, *p* < 0.05). Living with the biological father only was associated with higher odds of having multiple sexual partners (3.21, p < 0.10) and higher odds of occasional concurrent sexual partners (3.26, *p* < 0.10) among young males. Compared with their out-of-school counterparts, young males still enrolled in school were less likely to be sexually active in the last 12 months (0.33, *p* < 0.05) and less likely to have occasional concurrent sexual partners (0.57, p < 0.10), whereas young females still enrolled in school were more likely to be sexually active (2.25, *p* < 0.10) and less likely to use contraceptive consistently (0.36, p < 0.001).

**Conclusions:**

Reproductive health programs and interventions for preventing young people’s risky sexual behaviors in sub-Saharan African settings must take into account the protective effects of parent–child relationships and the significance of parental monitoring over time.

## Background

Young people’s involvement in risky sexual activities remains a concern in sub-Saharan Africa [1,2]. It is estimated that 71% of the adults and children newly infected worldwide in 2011 are from Sub-Saharan Africa, underscoring the importance of continuing and strengthening HIV prevention efforts in this region [3]. Identifying protective factors against risky sexual behaviors among young people is crucial for HIV prevention because these behaviors contribute greatly to the spread of HIV and other sexually transmitted infections. Almost 76% of young people infected in sub-Saharan Africa are females and female-to-male ratios of HIV infection range from 1.3 to 12 among youth aged 15–24 years [4]. HIV behavior change programs have chiefly been aimed at promoting delayed sexual initiation, decreasing overlapping sexual partnerships, and increasing correct and consistent use of condoms.

Research is just beginning to document the effects of parental influences on adolescent and reproductive health behaviors and outcomes in sub-Saharan Africa, using longitudinal data on parenting practices, family histories and transition to adulthood [5]. Parenting practices have been an influential factor for a wide range of behaviors including risky sexual behaviors among young people [6-8]. Family members and parents have been viewed as influential for children’s development and health outcomes, and parenting practices such as monitoring, support, and sexual communication between parents and adolescents, have been associated with children’s sexual behaviors [9,10].

Three main constructs of parenting practices have been consistently associated with children’s sexual behaviors: parent–child connectedness, parental monitoring, and parent–child communication on sexual and reproductive matters. There is evidence that child’s relations with father and mother are different, especially in respect of child’s gender.

### Parent–child connectedness

The connectedness has been conceptualized as a wide spectrum of social experiences, including the quality of a relationship, the degree of liking of an environment or a relationship, and the quality of performance in an environment or a relationship [11]. When focusing on parent–child interaction, “connectedness” typically refers to parental warmth, love, support, parent–child closeness, and child attachment to parent. Connectedness to parents is expected to facilitate the socialization of the child to parental values and guidance. Evidence from longitudinal and cross-sectional studies indicates that parental connectedness constitutes a protective factor against early sexual initiation, unwanted pregnancy or birth, and at-risk sexual activity in adolescence [8,10]. There is scanty evidence on the link between family connectedness and risky sexual behaviors such as unprotected sex and multiple sexual partners which remain key driving forces of HIV infection among young people [3].

Much of the research in sub-Saharan African settings has approached the parental connectedness within the confines of parental presence frequently studied in relation to the absent father [12,13] and to living arrangements [14-20]. Only three studies have investigated the effects of parent–child connectedness by using scales with different statements related to the perception of parental care, love, support, and the quality of the relationships. The first one pointed out that feeling connected to parents failed to emerge as a significant determinant of sexual behavior among the young in Zambia [21]. The second one revealed that a lower parent–child connectedness was positively associated with never having sex among secondary school students in Nigeria [22]. The third one showed that higher quality of parent–child relationships decreased significantly the risk of premarital intercourse among young people in Cameroon [23].

### Parental monitoring

Parental monitoring encompasses a set of correlated parenting practices aimed at structuring children’s homes, schools, and community, and at tracking their behavior in these environments [24]. It is conceptualized as being direct (the amount of time that the child is at home or in a public space with adult supervision) and indirect (the amount of knowledge that parents has of their children’s whereabouts, friends, or activities when they are not under their direct supervision). Indirect parental monitoring has been operationalized as a protective factor against children’s involvement in sexual risk-taking, although its effectiveness has been called into question as it mostly relied on children willing to disclose the information. Longitudinal studies in American settings suggest that direct and indirect parental monitoring has a protective effect on adolescent risky behaviors [10]. Some analyses with cross-sectional designs have indicated significant associations between low levels of indirect parental monitoring and greater odds of having had sexual intercourse among female and male adolescents in Burkina Faso, Ghana, and Uganda [14,20]. A recent study has revealed that a lower level of parental monitoring is significantly associated with an increase in the risk of premarital sexual intercourse among young Cameroonians [23].

### Parent–child communication on sexual and reprodutive matters

Communication about sexual and reproductive matters between parents and adolescents is probably the single parenting dimension for which the effects on adolescent sexual risk-taking remain unsettled due to methodological difficulties related to the temporal ordering of exposure and outcome variables. The expectation is that frequent and positive parent–child communication on such matters will lower the probability of sexual risk-taking by promoting more responsible adolescent behavior [9,10,25].

A recent review of studies of the effects of parent–child communication about sexuality and reproductive health matters on gender differences in children’s sexual behavior in sub-Saharan Africa has yielded conflicting results, although parent–child sexuality communication tended to be associated with reduction in adolescent risk-taking behaviors [26]. For instance, communication with parents on sex-related matters was significantly associated with increased likelihood of sexual activity among adolescent males in Malawi and adolescent females in Uganda [14], reduced hazard of sexual initiation among adolescent females in Kenya [27] and increased likelihood of regular condom use among Nigerian youths [28]; in Ghana, there was a strong negative relationship between parental monitoring and recent sexual activity for males and females, and limited effects of communication about sex-related matters [20]; in Tanzania, parent–child communication was not associated with the odds of sexual initiation in a longitudinal study of 12-14-year-old virgin primary school students who were followed up to 12 months [29]. Table 1 summarizes the main findings related to the associations between parenting practices and children’s sexual behavior in sub-Saharan African settings.

**Table 1:** Synopsis of extant literature on the association between parenting practices and sexual behavior of young people in sub-Saharan African societies

**Outcomes by parenting sub-constructs**	**Nature of the association**		
	**Protective association**	**Deleterious association**	**No association**
PARENT–CHILD CONNECTEDNESS			
**Parental presence**			
(9 *cross-sectional studies and 0 longitudinal study*)			
Having had sex in the last 12 months			14^a,*^;20^a,*^
Ever had sex	13^a,*, *x*^; 19^a,*^; 16 ^a,*, *x*^; 18^a, §^	17^a,§, *x*^	
Secondary sexual abstinence	16 ^a,*, *y*^		12 ^a,§^
Life time number of sexual partners			17^a,§^
Had > 1 partner in the last 3 months			12^a,§^_;_ 17^a,§^
Ever had an unwanted pregnancy	13^a,*, *x*^		
Having had sex in the last 4 weeks	13^a,*, *x*^		
Sexual initiation by age 17	15^a,§^		
**Multi-statements scale including statements related to**			
**support, love, care and quality of the relationships**			
(*3 cross-sectional studies and 0 longitudinal study*)			
Ever had sex	22^a,§^		21^a,§^
Life time number of sexual partners			21^a,§^
Had > 1 partner in the last 3 months			21^a,§^
Premarital sexual intercourse	23^a,§^		
PARENTAL MONITORING			
(*3 cross-sectional studies – 2 with the same source of data −*			
*and 0 longitudinal study*)			
Having had sex in the last 12 months	20^a,*^; 13^a,*^		
Premarital intercourse	23^a,§^		
PARENT–CHILD COMMUNICATION ON SEXUAL ISSUES			
(3 *cross-sectional studies and 1 longitudinal study*)			
Having had sex in the last 12 months		14^a,*^	
Premarital intercourse		23^a,§^	
Sexual initiation in early adolescence			28^b,*^
Timing of sexual initiation	26^a,*,*x*^		
Regular condom use	27^a,§^		

Notes: a: Cross-sectional study.

b: Longitudinal study.

*: Analyses based on a sample of adolescents (aged 19 years or younger).

**§**: Analyses based on a sample of adolescents and young people.

*x:* Effects for females.

*y*: Effects for males.

Our review is restricted to peer-reviewed publications involving parenting practices as main explanatory variables; no association means that no statistical difference was found.

The limited evidence identifying parent–child communication on sexual matters as a protective factor for adolescent sexual and reproductive well-being has not yet established whether such protective effect continue later in the life course of adolescents. Our study is designed to deepen the examination of links between sub-constructs of parent–child relationships, parental monitoring, parent–child communication and various measures of sexual risk-taking at different stages of development, by establishing whether early parenting practices influence sexual risk-taking later in the life course during adolescence and young adulthood. Such research is needed to better clarify the associations between parenting practices and sexual risk-taking, as well as the extent of changes and continuities in these associations during developmental stages in adolescence and young adulthood.

### Effects of early parenting practices on behaviors later in the life course

Our study seeks to unravel the effects of early parenting practices on children’s behaviors at different ages later in their lifecourse, and considers the extent of enduring effects of parenting practices on children’s sexual risk-taking behaviors in subsequent developmental transitions during adolescence and young adulthood. Our review of the extant literature identifies no study on this subject matter in the African context.

The development of cognitive and social capacities, which occurs during early adolescence, is supposed to make this developmental period an important window for shaping healthy attitudes and subsequent behaviors [30]. Studies which have substantiated the influence of parenting practices early in the life course of children on their sexual risk-taking behaviors in subsequent years have considered the pre-adolescence period [31-33] or the period from adolescence to early and late adulthood [34-36]. For instance, parental closeness and behavioral control during early adolescence (ages 12–14 years) have been linked to the number of sexual partners into early adulthood (ages 19–21 years) [34]. An important issue in addressing the effects of parenting practices early in the life course of children in subsequent developmnent stages is the identification of mechanisms operating to link early parenting practices to later risky sexual behaviors. Existing studies have considered the possibility that parental closeness and behavioral control would be associated with children’s sexual risk-taking through the delayed onset of sexual activity. Their findings suggest that monitoring may be more protective against sexual risk behavior for boys than girls [34-36]. Among females, parental closeness during early adolescence was associated with a fewer number of sexual partners during early adulthood directly, and indirectly through the sexual initiation during middle adolescence.

We consider the following three hypotheses:

*Hypothesis 1*. Parenting practices characterized by very good relationships, high level of monitoring, and sexual communication are associated with reduced sexual risk-taking;

*Hypothesis 2*. Parenting practices have enduring consequences for risk behaviors from adolescence into young adulthood.

*Hypothesis 3.* The enduring consequences of parenting practices for risk behaviors from adolescence into young adulthood are mediated by the delay in sexual initiation. Previous research in sub-Saharan African settings has provided empirical support for the linkage between the early onset of sexual activity and risky sexual behaviors later in life that allows us to speculate on this mediational effect [37,38].

## Methods

### Participants

The hypotheses enunciated above are tested using data on a representative and randomly sample of young people from the Cameroon Family and Health Survey (CFHS). Although the survey was carried out in 1996–1997, it possesses unique information in the African context on the timing of parenting practices for assessing the enduring consequences of parenting practices on sexual risk-taking of childen at different stages in their lifecourse and whether they vary by gender. The CFHS was designed with a theoretical approach that considers that risk and protective factors for health behaviors should be understood from a dynamic viewpoint, so as to take into account the influences of changes in an individual’s family, social, and environmental contexts over the life course. The survey was conducted in the Prefecture of Bandjoun, a semi-rural and developing area in the Western region of Cameroon. Bandjoun is located about 300 kilometres from the economic capital (Douala) and the political capital (Yaoundé). Bandjoun’s population was estimated at 51,624 inhabitants in 2005 [39]. Its inhabitants are mostly involved in the agricultural and retail sectors of the economy. Regarding the norms and sexual practices, this population tends to share negative opinions about the sexual activity among unmarried females and males and about an open discussion on sexual issues between parents and children [40]. Thus, parent–child communication on sexual issues is not an established practice in this population and may be influenced by the socioeconomic status of parents.

### Procedure

The CFHS is a survey of representative and randomly selected respondents from the population of Bandjoun aged 10 and older. The sampling procedure was initiated by an identification of the population composition of Bandjoun by sex (female and male) and by four age groups (10–14, 15–19, 20–49, and 50 or more), based on the information given by the latest (1987) Census of Population and Housing of Cameroon preceding the survey. All 75 localities forming the prefecture of Bandjoun were nested within 12 socio-sanitary areas created by the government of Cameroon. The CFHS used a self-weighted proportional sampling design. Households were randomly selected in these 12 areas, and respondents aged 10 years or older were randomly sampled from these households. Only one eligible respondent was randomly selected per household for interviewing, which ensured the absence of correlation between individuals at the household level. The process of random selection of respondents in selected households was performed until a representative sample by sex and four age groups was obtained for each socio-sanitary area. The final sample included 2,377 females and males aged 10 years old and older.

The data on parenting practices were collected in a section of the questionnaire entitled “family and residential environments”. Forty four questions were asked in order to have a full grasp of the characteristics of the family and residential contexts in which respondents’ lives were embedded. Each question was formulated in a retrospective life history approach, using predefined key moments in the life course to structure the respondents’ narratives. These key moments were: age 6, age 12, the moment of sexual initiation (for those who ever had sex) and the time of the survey. For each of these key moments if applicable, respondents had to provide information regarding various aspects of their family and residential environments including: the persons with whom they have lived, the attributes of parents/guardians who were responsible for their rearing, the quality of the relationship they have had with the parents/guardians, and the towns or localities they have lived in. These data do not constitute a “refined” reconstruction of respondents’ event histories (i.e. a reconstruction based on a year or a month scale). However, their retrospective longitudinal design constitutes a precious source of information to stimulate new venues of research in the sub-Saharan African context. Each of the selected key moments allows grasping experiences of individuals at different developmental stages in their life course.

### Ethical considerations and data access

The National Ethics Committee of Cameroon (Yaoundé, Cameroon) and the Ethics Committee of the University of Montreal (Montreal, Canada) provided ethical approval for the survey. Moreover, strict confidentiality was maintained about the identity of respondents due to the sensitivity of sexuality and reproductive health matters among unmarried youths. In fact, interviewers were instructed to respect respondents’ privacy during and after the interview. Specifically, interviews were not conducted in the presence of parents or guardians. The CFHS data are not openly accessible. The database, created and stored by the principal investigator, is available through written permission. More details about the survey are available elsewhere [41,42].

### Study sample

This study uses a subsample of unmarried and sexually active respondents aged 15–24 years old at the time of the survey. This subsample includes 447 individuals, with 220 (49.2%) males and 227 (50.8%) females. This subsample meets the sample size and power calculations criteria required for regression analyses and statistical inference [43].

### Measurements

*Sexual risk-taking* was measured using three survey items: the number of sexual partners in the last 12 months, the frequency of contraceptive use with sexual partners in the last 12 months and the tendency of having concurrent occasional partners. The questions were: “How many different sexual partners have you had during the last 12 months” – possible responses ranged from zero to six and more, “How often do you and/or partner … use a contraceptive method during sexual intercourse”, and “Do you usually have concurrent occasional sexual partners?” The question on contraceptive use with sexual partners did not specify the types of methods (i.e., condom versus other methods). However, it did specify use of a contraceptive method to avoid sexually transmitted diseases and/or unplanned pregnancy. The strategies used to reduce misreporting of sexual behaviors in the CFHS included: (1) stressing that the information to be collected during the interview will always remain strictly confidential as prescribed by law; and (2) making respondents aware of the importance of providing accurate information that may contribute to inform the design of effective sexual and reproductive health interventions.

Four dichotomous variables were created for the analysis: 1) sexually active in the last 12 months, 2) multiple partnerships in the last 12 months, 3) consistent use of contraceptive with sexual partners in the last 12 months, and 4) usually have concurrent occasional sexual partners. Because very few respondents reported systematic use of contraceptives, consistent use was defined as “systematic” or “regular” use. A good strategy of risk avoidance would, however, require systematic use of contraceptives.

#### Parenting practices

*Parent–child connectedness* was assessed using an item concerning the quality of the relationships with the parent/guardian. A question was asked about how the respondent perceived the quality of his/her relationship with his/her parent/guardian at each key moment. Possible responses ranged from 1 = “very good” to 4 = “difficult”. The variable was reversely coded to obtain a gradient with a higher score indicating a higher quality of parent–child relationships.

*Parental monitoring* was assessed using two items. The first item is the amount of parental knowledge regarding out-of-home activities of the respondents. A question was asked about how frequent the parents/guardians used to ask about their out-of-home activities at each key moment. The second item refers to the amount of parental approval regarding out-of-home activities. Respondents were asked a question about how frequent the parents/guardians agreed with their out-of-home activities at each key moment. For both questions, responses ranged from 1 = “a lot” to 5 = “never”. These two items were also reversely coded to create a gradient with a higher score indicating a higher level of parental monitoring.

*Parent–child communication* was assessed by four “yes or no” questions asking if respondents ever discussed with their parents/guardians at each key moment about puberty, sexual education, prevention of sexually transmitted infections and HIV/AIDS, and pregnancy prevention. These four items were summed; the scores ranged from 0 to 4. Cronbach’s α was .89 at age 12 and .84 at the time of the survey.

A summary of these measures of parenting practices is presented in Table 2.

**Table 2:** Items used to measure parenting dimensions in the Cameroon Family and Health Survey (CFHS)

**Dimension**	**Content**	**Measure**
PARENT–CHILD CONNECTEDNESS		
**Quality of parent/child relationships**	Perception of the quality of relationships with the parent or guardian at each key moment	4-point scale (ranged from 1 = “very good” to 4 = “difficult”)
PARENTAL MONITORING		
**Monitoring of out-of-home activities**	Amount of parental knowledge regarding out-of-home activities at each key moment: assessed by how often the parent or guardian asked about these activities	5-point scale (ranged from 1 = “a lot” to 5 = “never”)
**Approval of out-of-home activities**	Level of parental approval regarding out-of-home activities at each key moment: assessed by how often the parent or guardian agreed for these activities	5-point scale (ranged from 1 = “a lot” to 5 = “never”)
PARENT–CHILD COMMUNICATION ON PUBERTY, SEXUALITY, HIV/AIDS, PREGNANCY		
**4 items** (Cronbach’s α was .89 at age 12 and .84 at the time of the survey)	Discussion about puberty	“Yes” or “No” question
	Discussion about general sexual matters	“Yes” or “No” question
	Discussion about HIV/AIDS prevention	“Yes” or “No” question
	Discussion about pregnancy prevention	“Yes” or “No” question

Notes: Parenting dimensions have been assessed from respondents’ perspective.

The key moments included: age 6, age 12, and the time of survey.

#### Other variables

A number of commonly used family background variables are available in the CFHS, and we used them in our models since they have been identified in prior research as aspects of parenting practices [24]. These variables include: the family structure and the parent/guardian socioeconomic characteristics. The family structure was measured by asking: “Were you living with your biological parents at …? (each time point)” and “Who were you living with in the household at …? (each time point) ». We created a single variable indicating if respondents were living with both biological parents, the biological mother only, the biological father only, other relatives or with uncorrelated residents. Variables capturing the socioeconomic status of parent/guardian include education -- whether the parent/guardian had at least a primary level of education, employment -- whether the parent/guardian had a paid job, and marital status - whether the parent/guardian was married. Note that the information used to create these two variables was collected from the respondents.

Gender and age were also included in the analyses as they may influence the association between parenting practices and respondents’ sexual risk-taking. Gender was self-reported (coded 0 for females and 1 for males). Age corresponds to the respondents’ responses in completed years. Other variables added in the models included current school enrolment status and religious affiliation which are known correlates of young people’s sexual risk-taking in sub-Saharan Africa [12,21].

### Data analysis

Logistic regressions are performed to estimate the odds and standard errors of having risky behaviors. Two models are used. The first model tests the effects of parenting practices at young ages (assessment at the time of the survey) as well as the effects of parenting practices during early adolescence (assessment at age 12). The other variables are also fitted in the model. As parenting practices early in the children’s life course are expected to be indirectly associated with sexual risk-taking at young ages through delayed onset of sexual activity, we use a second model that includes a variable related to whether or not the respondent had initiated sex by age 15.

## Results

Table 3 presents the descriptive analysis by sex, for unmarried and sexually active respondents who were aged 15–24 years at the survey date. The mean age of respondent was 19.3 years, and the majority of respondents were aged 15–19 years (59.6% and 58.2% among male and female respondents, respectively). At the survey date, 49.1% of males compared with only 43.2% of females were enrolled in school, and 90.8% of respondents are Christians. Over 80% (87.3% males and 83.3% females) of them reported to have been sexually active in the last 12 months. More males (41.4%) than females (10.6%) reported to have had concurrent occasional sexual partners. Although the percentage of young people who reported having had multiple sexual partners is lower for females (15.9%) than males (42.2%), the proportions of females and males who reported a consistent use of contraceptives with partner in the last 12 months are similar. Males and females reported high levels of parental monitoring, moderate quality of parent–child connectedness and low levels of parent–child communication on sexual matters during early adolescence and young adulthood. Young females reported significantly more sexual communication with their parents or guardians than young males during early adolescence and in young ages.

**Table 3:** Descriptive results

**Variables**	**Males**	**Females**	**Total**
	**(n = 220)**	**(n = 227)**	**(n = 447)**
*Sexual risk-taking*			
Sexually active in the last 12 months (% yes)	87.3	83.3	85.2
Multiple partnerships in the last 12 months (% yes)	42.2	15.9	29.1
Consistent use of contraceptive with partners in the last 12 months (% yes)	29.2	30.7	29.9
Used to having concurrent occasional sexual partners (% yes)	41.4	10.6	25.7
*Parenting dimensions in young ages(assessment at the time of the survey)*			
Quality of parent/child relationships (range, 1–4)	2.09(±0.79)	2.11(±0.86)	2.10(±0.83)
Knowledge of out-of-home activities (range, 1–5)	2.29(±1.44)	3.06(±1.48)	2.68(±1.51)
Approval of out-of-home activities (range, 1–5)	3.19(±1.41)	3.05(±1.31)	3.13(±1.36)
Parent–child communication on sexual issues (range, 0–4)	0.88(±0.88)	1.18(±1.47)	1.04(±1.42)
*Parenting dimensions during early adolescence (assessment at age 12)*			
Quality of parent/child relationships (range, 1–4)	1.95(±0.73)	1.99(±0.76)	1.98(±0.75)
Knowledge of out-of-home activities (range, 1–5)	3.72(±1.35)	3.88(±1.23)	3.80(±1.29)
Approval of out-of-home activities (range, 1–5)	3.13(±1.26)	3.09(±1.21)	3.11(±1.23)
Parent–child communication on sexual issues (range, 0–4)	0.30(±0.92)	0.67(±1.26)	0.47(±1.12)
*Mediating variable*			
Early sexual debut (sexual initiation by age 15)	45.0	31.3	38.0
*Other variables*			
Family structure^‡^			
Both biological parents	41.8	48.0	44.9
Biological mother only	13.6	16.3	15.0
Biological father only	5.0	7.5	6.3
Other relatives	19.1	18.1	18.6
Uncorrelated residents	20.5	10.1	15.2
Parent/guardian socioeconomic characteristics^‡^			
Parent/guardian has at least a primary level of education	68.2	85.0	76.7
Parent/guardian has a paid job	31.8	40.9	36.6
Parent/guardian is married	65.5	75.8	70.7
Respondent’ age			
15-19	59.6	58.2	58.8
20-24	40.4	41.8	41.2
Currently attending school			
Yes	49.1	43.2	46.1
No	50.9	56.8	53.9
Religious affiliation			
Christian	85.5	96.0	90.8
Others	14.5	4.0	9.2

Notes: ^‡^ At the time of the survey ± Standard deviation.

All scales are scored such that a higher value indicates higher level of the construct.

### Influences of parenting practices on sexual risk-taking during adolescence and young adulthood

Multivariate results of the effects of parenting practices on sexual risk-taking behaviors among males and females are presented in Table 4 and Table 5, respectively.

**Table 4:** Results from the logistic regression analysis identifying associations between parenting practices and the odds of sexual risk-taking for MALES (n = 220)

**Variable**			**Odds ratio (95% Confidence Intervals)**					
	**Sexually active (n = 220)**		**Multiple partnerships (n = 220)**		**Consistent use of contraceptive**^**© **^**(n = 192)**		**Occasional concurrent sexual partners (n = 220)**	
	**Model 1**	**Model 2**	**Model 1**	**Model 2**	**Model 1**	**Model 2**	**Model 1**	**Model 2**
*Parenting dimensions (assessment at the time of the survey)*								
Quality of parent/child relationships	0.67	0.67	0.63**	0.64**	0.65	0.66	0.79	0.81
	(0.31-1.43)	(0.32-1.39)	(0.40-0.98)	(0.41-0.99)	(0.39-1.08)	(0.39-1.09)	(0.52-1.19)	(0.53-1.21)
Knowledge of out-of-home activities	0.77*	0.78*	1.12	1.08	1.01	1.01	1.04	1.02
	(0.57-1.04)	(0.58-1.05)	(0.91-1.39)	(0.86-1.36)	(0.78-1.30)	(0.78-1.29)	(0.84-1.27)	(0.82-1.25)
Approval of out-of-home activities	0.81	0.81	0.98	0.98	0.99	1.01	1.12	1.12
	(0.55-1.19)	(0.56-1.19)	(0.77-1.22)	(0.78-1.23)	(0.77-1.28)	(0.77-1.28)	(0.89-1.41)	(0.89-1.41)
Parent–child communication on sexual issues	1.33	1.33	1.25*	1.27*	0.88	0.88	1.24*	1.25*
	(0.83-2.11)	(0.83-2.12)	(0.96-1.62)	(0.97-1.64)	(0.67-1.15)	(0.67-1.15)	(0.96-1.60)	(0.97-1.61)
*Parenting dimensions during early adolescence (assessment at age 12)*								
Quality of parent/child relationships	1.24	1.24	0.99	1.00	0.84	0.84	1.09	1.10
	(0.64-2.38)	(0.64-2.37)	(0.60-1.63)	(0.61-1.65)	(0.51-1.37)	(0.51-1.37)	(0.69-1.72)	(0.69-1.74)
Knowledge of out-of-home activities	1.18	1.19	0.91	0.92	0.93	0.94	0.83	0.84
	(0.82-1.70)	(0.82-1.70)	(0.71-1.16)	(0.72-1.17)	(0.71-1.22)	(0.72-1.23)	(0.66-1.04)	(0.67-1.05)
Approval of out-of-home activities	1.17	1.17	0.96	0.96	1.12	1.13	0.92	0.92
	(0.79-1.72)	(0.79-1.72)	(0.74-1.24)	(0.74-1.24)	(0.84-1.48)	(0.84-1.50)	(0.72-1.18)	(0.71-1.19)
Parent–child communication on sexual issues	1.46	1.46	1.01	0.99	0.93	0.93	0.95	0.95
	(0.59-3.64)	(0.58-3.66)	(0.67-1.51)	(0.65-1.51)	(0.63-1.36)	(0.63-1.36)	(0.67-1.36)	(0.66-1.35)
Early sexual debut (sexual initiation by age 15)		0.93		1.72		1.13		1.36
		(0.41-2.13)		(0.88-3.37)		(0.54-2.33)		(0.74-2.49)
Family structure^‡^ (ref: Both biological parents)								
Biological mother only	1.67	1.68	1.93	1.96	0.62	0.61	1.21	1.22
	(0.29-9.37)	(0.29-9.54)	(0.71-5.26)	(0.68-5.61)	(0.18-2.00)	(0.18-1.99)	(0.46-3.18)	(0.45-3.24)
Biological father only	2.12	2.12	3.21*	3.27 *	1.14	1.12	3.26*	3.26*
	(0.30-4.75)	(0.29-5.07)	(0.77-3.24)	(0.82-2.99)	(0.27-4.74)	(0.27-4.64)	(0.87-2.20)	(0.88-2.08)
Other relatives or unrelated coresidents	3.78*	3.78*	0.69	0.62	1.77	1.74	1.03	0.98
	(0.76-7.90)	(0.80-7.75)	(0.27-1.74)	(0.23-1.67)	(0.62-5.08)	(0.60-5.06)	(0.44-2.37)	(0.42-2.31)
Uncorrelated residents	0.71	0.71	2.56*	2.40*	0.62	0.60	1.62	1.55
	(0.14-3.67)	(0.13-3.73)	(0.99-6.61)	(0.92-6.24)	(0.18-1.81)	(0.20-1.80)	(0.65-4.04)	(0.62-3.89)
Parent/guardian socioeconomic characteristics^‡^								
Parent/guardian has a primary education (ref: No)	0.72	0.72	0.38*	0.34**	0.86	0.84	0.75	0.72
	(0.13-3.77)	(0.13-3.90)	(0.13-1.05)	(0.12-0.97)	(0.23-3.09)	(0.23-3.03)	(0.28-1.98)	(0.27-1.88)
Parent/guardian has a paid job (ref: No)	0.85	0.85	1.29	1.37	1.16	1.17	1.34	1.37
	(0.31-2.34)	(0.31-2.35)	(0.56-2.97)	(0.59-3.19)	(0.48-2.83)	(0.48-2.85)	(0.64-2.80)	(0.66-2.86)
Parent/guardian is married (ref: No)	1.42	1.41	1.50	1.52	0.54	0.54	0.83	0.83
	(0.31-6.47)	(0.31-6.48)	(0.50-4.41)	(0.51-4.49)	(0.15-1.88)	(0.15-1.87)	(0.31-2.18)	(0.31-2.15)
Respondent’ age (ref: 15–19)	0.96	0.95	2.02**	2.21**	0.82	0.83	1.62	1.71*
	(0.41-2.29)	(0.40-2.26)	(1.06-3.83)	(1.13-4.29)	(0.40-1.67)	(0.40-1.71)	(0.88-2.97)	(0.92-3.16)
Currently attending school (ref: No)	0.33**	0.32**	0.60	0.63	0.65	0.66	0.57*	0.59*
	(0.11-0.98)	(0.10-1.00)	(0.28-1.29)	(0.29-1.36)	(0.28-1.47)	(0.39-1.09)	(0.28-1.11)	(0.30-1.16)
Religious affiliation (ref: Christian)	0.68	0.69	1.20	1.17	0.97	0.97	1.26	1.23
	(0.15-3.13)	(0.15-3.11)	(0.45-3.21)	(0.44-3.10)	(0.38-2.47)	(0.38-2.43)	(0.55-2.88)	(0.53-2.82)

Notes: ^‡^ At the time of the survey ^©^Among those who had at least one sexual partner.

All scales are scored such that a higher value indicates higher level of the construct.

Model 1: Parenting variables + controls for family structure, parent/guardian socioeconomic characteristics, age, schooling status and religious affiliation;

Model 2: Parenting variables + controls for early sexual debut, family structure, parent/guardian socioeconomic characteristics, age, schooling status and religious affiliation.

Level of significance: * p < 0.1; ** p < 0.05; *** p < 0.001.

**Table 5:** Results from the logistic regression analysis identifying associations between parenting practices and the odds of sexual risk-taking for FEMALES (n = 227)

**Variable**	**Odds ratio (95% Confidence Intervals)**							
	**Sexually active (n = 227)**		**Multiple partnerships**		**Consistent use of contraceptive**^**©**^		**Occasional concurrent sexual partners**	
			**(n = 227)**		**(n = 189)**		**(n = 227)**	
	**Model 1**	**Model 2**	**Model 1**	**Model 2**	**Model 1**	**Model 2**	**Model 1**	**Model 2**
*Parenting dimensions (assessment at the time of the survey)*								
Quality of parent/child relationships	0.52*	0.54*	0.64*	0.60*	0.90	0.88	0.51*	0.46**
	(0.26-1.02)	(0.28-1.04)	(0.36-1.11)	(0.34-1.05)	(0.54-1.50)	(0.52-1.48)	(0.26-1.02)	(0.23-0.88)
Knowledge of out-of-home activities	1.04	1.03	1.31*	1.32*	1.09	1.10	0.92	0.91
	(0.79-1.35)	(0.78-1.34)	(0.94-1.81)	(0.95-1.81)	(0.85-1.40)	(0.86-1.40)	(0.68-1.24)	(0.67-1.23)
Approval of out-of-home activities	1.35*	1.36*	0.90	0.90	1.01	0.99	1.15	1.12
	(0.98-1.87)	(0.98-1.86)	(0.63-1.30)	(0.63-1.29)	(0.73-1.36)	(0.72-1.36)	(0.74-1.78)	(0.74-1.70)
Parent–child communication on sexual issues	1.23	1.24	1.21	1.18	0.91	0.89	1.22	1.20
	(0.91-1.64)	(0.92-1.67)	(0.86-1.70)	(0.83-1.67)	(0.67-1.21)	(0.65-1.21)	(0.79-1.86)	(0.79-1.81)
*Parenting dimensions during early adolescence (assessment at age 12)*								
Quality of parent/child relationships	1.32	126	0.93	0.99	0.64	0.66	1.35	1.54
	(0.65-2.67)	(0.63-2.51)	(0.46-1.87)	(0.48-2.06)	(0.33-1.22)	(0.34-1.26)	(0.60-3.02)	(0.68-3.48)
Knowledge of out-of-home activities	1.40**	1.39**	0.87	0.88	0.99	0.99	0.97	0.99
	(1.03-1.89)	(1.01-1.89)	(0.61-1.25)	(0.61-1.28)	(0.74-1.33)	(0.74-1.32)	(0.67-1.39)	(0.67-1.46)
Approval of out-of-home activities	0.82	0.79	0.82	0.83	0.94	0.95	0.70	0.73
	(0.59-1.13)	(0.57-1.11)	(0.54-1.22)	(0.55-1.24)	(0.67-1.32)	(0.67-1.33)	(0.46-1.08)	(0.47-1.12)
Parent–child communication on sexual issues	0.86	0.88	0.77	0.76	0.88	0.88	0.66	0.63
	(0.61-1.21)	(0.61-1.22)	(0.45-1.31)	(0.45-1.27)	(0.64-1.21)	(0.64-1.21)	(0.34-1.27)	(0.34-1.15)
Early sexual debut (sexual initiation by age 15)		0.63		2.03*		1.29		2.69*
		(0.28-1.39)		(0.85-4.87)		(0.59-2.84)		(0.92-7.82)
Family structure^‡^ (ref: Both biological parents)								
Biological mother only	0.81	0.83	1.26	1.22	0.78	0.75	0.18*	0.17
	(0.24-2.68)	(0.25-2.74)	(0.31-5.08)	(0.29-5.03)	(0.25-2.40)	(0.24-2.30)	(0.02-1.37)	(0.02-1.39)
Biological father only	3.49	3.64	1.80	1.88	0.33*	0.32*	5.92**	6.73**
	(0.66-8.41)	(0.64-8.44)	(0.31-10.12)	(0.32-10.78)	(0.09-1.09)	(0.09-1.07)	(1.38-5.43)	(1.57-8.89)
Other relatives or unrelated co residents	2.29	2.30	1.19	1.11	0.55	0.53	0.53	0.50
	(0.65-8.01)	(0.66-7.91)	(0.36-3.90)	(0.32-3.77)	(0.20-1.48)	(0.19-1.46)	(0.19-2.20)	(0.11-2.10)
Uncorrelated residents	1.55	1.51	3.29	3.91	0.85	0.91	0.63	0.78
	(0.39-6.10)	(0.36-6.23)	(0.79-9.70)	(0.87-9.42)	(0.24-2.96)	(0.25-3.21)	(0.15-2.60)	(0.16-3.81)
Parent/guardian socioeconomic characteristics^‡^								
Parent/guardian has a primary education (ref: No)	1.34	1.36	1.47	1.55	0.49	0.50	0.81	0.83
	(0.39-4.59)	(0.38-4.91)	(0.31-6.81)	(0.29-8.24)	(0.15-1.58)	(0.15-1.60)	(0.16-4.05)	(0.14-4.69)
Parent/guardian has a paid job (ref: No)	0.73	0.72	0.60	0.56	1.21	1.22	0.52	0.48
	(0.33-1.56)	(0.33-1.55)	(0.24-1.50)	(0.21-1.42)	(0.57-2.54)	(0.58-2.58)	(0.18-1.48)	(0.16-1.42)
Parent/guardian is married (ref: No)	0.64	0.66	0.62	0.58	1.48	1.47	0.78	0.77
	(0.18-2.25)	(0.17-2.39)	(0.19-2.04)	(0.17-1.98)	(0.55-3.99)	(0.54-3.96)	(0.22-2.72)	(0.21-2.78)
Respondent’ age (ref: 15–19)	0.43**	0.38**	1.61	1.79	1.12	1.17	1.08	1.26
	(0.19-0.92)	(0.17-0.84)	(0.64-4.05)	(0.70-4.51)	(0.53-2.38)	(0.55-2.48)	(0.37-3.13)	(0.42-3.71)
Currently attending school (ref: No)	2.25*	2.27*	1.15	1.11	0.36***	0.35***	2.15	2.13
	(0.96-5.25)	(0.99-5.21)	(0.47-2.78)	(0.46-2.65)	(0.17-0.71)	(0.17-0.70)	(0.79-5.77)	(0.80-5.69)
Religious affiliation (ref: Christian)	0.73	0.75	0.26	0.22	1.16	1.14	0.52	0.41
	(0.07-7.43)	(0.07-8.08)	(0.03-1.67)	(0.03-1.35)	(0.24-5.49)	(0.23-5.49)	(0.04-6.11)	(0.04-3.92)

Notes: ^‡^ At the time of the survey ^©^Among those who had at least one sexual partner.

All scales are scored such that a higher value indicates higher level of the construct.

Model 1: Parenting variables + controls for family structure, parent/guardian socioeconomic characteristics, age, schooling status and religious affiliation;

Model 2: Parenting variables + controls for early sexual debut, family structure, parent/guardian socioeconomic characteristics, age, schooling status and religious affiliation.

Level of significance: * p < 0.1; ** p < 0.05; *** p < 0.001.

After controlling for all measured covariates except early sexual debut in each of the two tables (Model 1), a number of findings emerge for young males and females. Young males who reported a higher quality of relationships with their parents/guardians early in their life course were significantly less likely to have had multiple sexual partners in the last 12 months (0.63, *p* < 0.05). Parental knowledge of out-of-home activities of young males was associated with reduced odds of sexual activity (0.77, p < 0.10). Young males who have had discussion about sexual issues with their parents had higher odds of having multiple partnerships (1.25, *p* < 0.10) and higher odds of having occasional concurrent sexual partners (1.24, p < 0.10). Family structure, parent/guardian educational level, respondent’s age and enrollment status exhibited significant associations with sexual risk-taking behaviors among young males. Living with the biological father only was associated with higher odds of having multiple sexual partners (3.21, p < 0.10) and of occasional concurrent sexual partners (3.26, *p* < 0.10) among young males. Compared with young males who were living with both their biological parents, those who were living with uncorrelated residents were more likely to have multiple sexual partners (2.56, *p* < 0.10) and those who co-residing with other relatives or unrelated co-residents were more likely to be sexually active in the last 12 months (3.78, p < 0.10). Young males who were raised by a parent/guardian with at least a primary education had reduced odds of having multiple sexual partners (0.38, *p* < 0.10). Compared with their out-of-school counterparts, young males who were still enrolled in school were less likely to be sexually active in the last 12 months (0.33, *p* < 0.05) and less likely to have occasional concurrent sexual partners (0.57, p < 0.10). Compared with adolescent males aged 15–19 years, older males aged 20–24 years were significantly more likely to have multiple sexual partners (2.02, p < 0.05). These results and their significance levels among young males remain unchanged after controlling for the timing of sexual debut (Model 2).

As regards young females, having very good relationships with their parents significantly decreased their odds of sexual risk-taking: the higher the quality of parent–child relationships, the lower the odds of being sexually active in the last 12 months (0.52, *p* < 0.10), of having multiple sexual partners in the last 12 months (0.64, p < 0.10) and of having occasional sexual partners (0.51, *p* < 0.05). In contrast to young males, parental knowledge of out-of-home activities of young females was associated with increased odds of sexual activity (1.31, p < 0.10) and young females whose parents approved of their out-of-home activities had higher odds of being sexually active in the last 12 months (1.35, *p* < 0.10) than other young females. Family structure is another significant factor affecting sexual risk-taking among young females: those who were living with their biological father only were significantly more likely to have occasional sexual partners (5.92, p < 0.05) and less likely to use contraceptive in a consistent fashion. In contrast to findings among males, young females who were still enrolled in school were more likely to be sexually active in the last 12 months (2.25, *p* < 0.10) and less likely to use contraceptive consistently (0.36, p < 0.001) compared with their out-of-school female counterparts. As expected, adolescent females aged 15–19 years are significantly less likely to be sexually active (0.43, p < 0.05) compared with older females aged 20–24 years. Early sexual debut was associated with multiple sexual partnerships (2.03, p < 0.10) and occasional concurrent sexual partners (2.69, p < 0.10) in the last 12 months. However, controlling for the timing of sexual initiation changed little these findings reported here.

### Enduring influences of parenting practices on children’s sexual risk-taking behaviors during adolescence and young adulthood

We found that parenting practices during early adolescence were not significantly associated with sexual risk-taking behaviors in young ages among males. Our results show however that among females, parental monitoring during early adolescence was associated with odds of being sexually active in the last 12 months that were 40% higher that the odds among females who did not have such parenting (1.40, *p* < 0.05). These findings are not affected by a control for the timing of the onset of sexual activity, and reject the hypothesis related to a mediating effect of early sexual initiation.

## Discussion

The purpose of this study was to investigate the associations between parenting practices and sexual risk-taking in Cameroon. As reported by previous studies conducted in Cameroon, the percentages of young people who are involved in risky sexual behaviors are generally higher among males that among females [2,5]. Our study showed that roughly an equal proportion of females and males reported a consistent use of contraceptive with a partner in the last 12 months preceding the survey date. Young females reported significantly more sexual communication with their parents/guardians in young ages and during early adolescence than did young males. This finding is consistent with previous studies which have highlighted this discrepancy [14,44]. One explanation is that parents may have perceived that their unmarried daughters were more vulnerable to social and health consequences of sexual activity, and engaged early discussions on sexual matters with them.

Contrary to the study by Magnani et al. [21] in urban Zambia which found no association between the quality of parent–child relationships and the number of sexual partners, we found that a very good parent–child relationship is protective against multiple partnerships among males and among females. A very good parent–child relationship is also protective against sexual initiation and occasional concurrent sexual partnerships among females in young ages. As regards the influences of parental monitoring, both parental knowledge of out-of-home activities and parental approval of out-of-home activities have no significant association with sexual risk-taking behaviors among males. Among females however, parental knowledge of out-of-home activities is associated with an increase in the odds of multiple partnerships in young ages and parental approval of out-of-home activities is associated with an increase in the odds of sexual activity. These results for females contradict those obtained by Biddlecom et al. [14] showing protective effects of parental monitoring on sexual activity in a sample of adolescent females in urban settings. This difference in findings between our study and the study by Biddlecom et al. [14] may hinge in part on differences in sheer numbers involved or the urban sample in the latter study in contrast to the small and predominantly rural sample in our study.

A risk association between parent–child communication on sexual matters and sexual activity in young ages among males and females is found as reported in previous studies [14,23]; in addition, we also found a risk association between parent–child communication on sexual issues and multiple partnerships and occasional concurrent sexual partnerships among males and females. Indeed, several factors complicate an understanding of how parent–child communication on sexual issues could contribute to deter young people’s involvement in risky sexual behaviors. Thus far, it has been found that parents do not constitute a favored or highly used source for information on sex-related matters in several settings [45,46]. Researchers’ interpretations of the low levels of parent–child communication emphasize three main aspects. First, the transmission of information on cultural norms of sexual conduct by parents is not a traditional practice in most African settings [2,47]. Initiation rites and ceremonies have traditionally served as community plattforms through which information related to puberty, pregnancy risks, personal hygiene, contraceptive use were transmitted to individuals [2,5,48]. Second, low levels of parent–child communication on sexual issues may be explained by the sharing of child-rearing responsibilities between the parents and other family members from the nuclear or the extended family unit [2,5,49]. Third, the formal nature of parent–child relationship is thought to be a factor that restrains the ease of discussion on sexual issues [2,50]. Regardless of these facts, more investigation is needed to determine aspects of parent–child communication such as the timing, the content, the openness or the frequency of communication which could be influential in young people sexual risk-taking in sub-Saharan African settings.

Our study yields no convincing evidence in support of the enduring effects of parenting practices during early adolescence on children’s sexual risk-taking behaviors during adolescence and young adulthood. No association was found among males. Among females, parental knowledge of out-of-home activities during early adolescence appeared to be associated with an increase in the odds of sexual activity at young ages. Parent–child discussion on sexual issues during early adolescence was associated with lower odds of having multiple sexual partners and lower odds of having occasional concurrent sexual partners in young ages among females, even if such association was not statistically significant. A further investigation of the extent and enduring consequences of parenting practices and parent–child sexual communication on risky sexual behaviors on emerging and young adult is needed in the African context.

### Limitations

Although this study advances the state of knowledge on the effects of parenting practices on sexual risk-taking behaviors during adolescence and young adulthood in Africa, there are some potential limitations that should be noted. First, the studied sample is relatively small (n = 447). Second, the measures of parenting practices have been assessed solely from the respondents’ perspective. Previous research using parent–child dyads have shown that there are some inconsistencies between parents and children reports, suggesting that children reports which reflect their reading of their relationships with their parents may not reflect actual parental practices. Moreover, the retrospective design used to collect the data that we analyzed here may have affected the quality of the respondents’ reports. Retrospective data are considered to be subjected to recall errors. This problem is particularly pervasive when the recall events happened in a distant past. Because information was gathered by using key time points (i.e. age 12, along with the time of the survey), the likelihood that respondents associated their parenting experiences with the correct time period is expected to be quite high. Finally, this study is based on quite old data collected in 1996–1997; its findings while important may not necessarily reflect prevailing current conditions in the studied localities of Cameroon.

Notwithstanding these shortcomings, our study contributes to the search for evidence on effects of parenting practices on children’s risky sexual behavior in African societies. Findings that very good relations between parents and children are protective against having multiple sexual partners among young males and against occasional concurrent sexual partnerships among young females are indeed robust.

## Conclusions

This study provides supporting evidence to the gender development theory positing that parenting practices is differentially linked to risk-taking behavior of males and females. More studies are needed to inform programs aiming at reducing the levels of sexual risky behaviors among young people in sub-Saharan African societies by involving parents. To date, a number of programs have been implemented in numerous settings in order to identify and enhance the parenting facets considered to act as protective factors against sexual risk-taking [51] without a significant base of evidence. Because they constitute a crucial part in their social environment and daily interactions, family members and parents in particular, are viewed as influential actors for children’s development and health outcomes. Our results suggest that the quality of parent–child relationships may be a dimension that is worth strenghtening through programmatic efforts. Parents should be aware of the importance of having close relationships with their children during their developmental stages through adolescence, emerging adulthood and young adulthood.

## Competing interests

The authors declare that they have no competing interests.

## Authors’ contributions

All authors (EMS, BKD) participated in the study design and the conception of research questions, and equally contributed to this paper. BKD was the principal investigator of Cameroon Adolescent Reproductive Health Program (CAREH) which consisted of two components, including Intervention and Research. EMS led the analysis and drafted the paper. BKD supervised the process and reviewed each draft of the paper. Both authors read and approved the final manuscript.

## Pre-publication history

The pre-publication history for this paper can be accessed here:

http://www.biomedcentral.com/1471-2458/13/616/prepub
